# Association of asthma control, treatment, adherence, lung function, and allergy sensitization with depressive symptoms and quality of life in a real-life setting

**DOI:** 10.3389/fpsyg.2026.1882738

**Published:** 2026-07-31

**Authors:** Gandhi F. Pavón-Romero, Yvonee Flores-Medina, Esteban Payan-Espindola, Ximena Cabrera-González, Jessica Cruz-Pérez, Ramírez-Jiménez Fernando, Lenin Pavón, Luis M. Teran

**Affiliations:** 1 Department of Immunogenetics and Allergy, National Institute of Respiratory Diseases. Ismael Cosío Villegas. Mexico City, Mexico; 2 Schizophrenia Clinic, National Institute of Psychiatry, Ramón de la Fuente Muñiz, Mexico City, Mexico; 3 Laboratory de Psicoinmunology, National Institute of Psychiatry, Ramón de la Fuente Muñiz, Mexico City, Mexico

**Keywords:** adherence, asthma, depressive symptoms, lung function, quality of life

## Abstract

**Introduction:**

Asthma is a chronic inflammatory respiratory disease that is frequently associated with emotional distress and impaired quality of life. However, how clinical factors, such as treatment, adherence, lung function, and allergy sensitization, relate to depressive symptoms remains unclear. In this study, we examined the associations among asthma control, treatment, adherence, lung function, allergy sensitization, somatic and cognitive-affective depressive symptoms, and quality of life in a real-life clinical setting.

**Methods:**

This cross-sectional study included adults with asthma attending tertiary pneumology and allergy services, and their asthma symptoms were evaluated using the standardized measures of the Asthma Control Test, GINA 2022 (treatment), Test of Adherence to Inhalers, lung function, skin prick test for allergy sensitization, PHQ-9 (depressive symptoms), Asthma Quality of Life Questionnaire, serum eosinophils and IgE levels.

**Results:**

Of 102 asthma patients, 51% of participants scored more than 5 points on the PHQ-9, indicating the presence of depression. Poor asthma control and lower adherence scores were significantly associated with higher depressive symptoms (*r*_*s*_ = −0.36, *p*< 0.001; *r*_*s*_ = 0.41, *p*< 0.001) and poorer quality of life (*r*_*s*_ = 0.50, *p*< 0.001), whereas objective lung function like reduced forced vital capacity (*r*_*s*_ = 27, *p* = 0.05) was weakly associated with depressive symptoms. Eosinophils count (*r*_*s*_ = 0.02), IgE (*r*_*s*_ = 0.06) or allergy sensitization (*r*_*s*_ = 0.08) markers showed nonsignificant associations with emotional outcomes.

**Conclusion:**

Emotional distress and quality of life are more strongly related to patients’ perceived control of their disease and adherence behaviors than to objective physiological markers. Thus, psychological and self-management strategies should be integrated into routine asthma care.

## 1 Introduction

Asthma is a heterogeneous disease that is typically characterized by chronic airway inflammation, which impairs breathing (Global Initiative for Asthma, 2022), and a history of respiratory symptoms, including wheezing, shortness of breath, chest tightness, and cough, which vary in intensity and frequency; these symptoms lead to variable expiratory airflow limitations (Global Initiative for Asthma, 2022).

It affects individuals of all ages and is a significant global health burden, especially in low- and middle-income countries. Asthma attacks can be triggered by allergens, tobacco smoke, exercise, and intense emotions (Global Initiative for Asthma, 2022). According to the Global Asthma Report, the global prevalence of asthma is 9.1% among children, 11.0% among adolescents, and 6.6% among adults (Institute for Health Metrics and Evaluation, 2023). The National Institute of Respiratory Diseases (INER) in Mexico reported that approximately 8.5 million people are affected by asthma (Asthma).

Asthma is often diagnosed in infancy and is monitored throughout a patient’s lifetime. It is estimated that 50% of children with asthma have poorly controlled symptoms (Sullivan et al., 2019). For these children, asthma is a leading cause of school absences and hospital visits (Global Initiative for Asthma, 2022). It also limits their physical activity and can negatively affect their self-esteem (Gibson-Scipio et al., 2023). These factors can negatively affect a child’s mental health, leading to anxiety and depression. Additionally, children and young adults with medical conditions often experience psychosocial stressors, such as social stigma, isolation, and bullying, which can severely affect their self-esteem and mental wellbeing (Liccardi et al., 2024). Chronic illnesses such as asthma trigger a sustained stress response, leading to changes in brain chemistry and hormonal imbalances. These factors significantly contribute to the development of anxiety and depression, as well as of low quality of life (Ciprandi et al., 2015; Gao et al., 2015; Lu et al., 2018; Sanna et al., 2014; Sastre et al., 2018).

Depressive symptoms and asthma affect patients’ quality of life; however, how factors such as treatment, patients’ adherence to treatment, or lung function contribute to emotional distress in these patients is still unclear. In this study, we aimed to describe the relationships among asthma control, treatment, adherence, lung function, and allergy sensitization and somatic or cognitive-affective depressive symptoms and quality of life in patients with asthma in a real-life setting during the first allergy medical consultation.

## 2 Materials and methods

### 2.1 Study design

This cross-sectional study included Mexican patients with asthma who attended the allergy clinic of the Department of Immunogenetics and Allergy (DIA) of the National Institute of Respiratory Diseases, Ismael Cosío Villegas. We enrolled patients over 18 years of age of any gender with a confirmed diagnosis of asthma (symptoms-cough, shortness of breath, wheezing, chest tightness, and a positive reversibility test-increase of 12% in FEV_1_ compared to the baseline measurement after administration of 400 μg of salbutamol).

**FIGURE 1:**
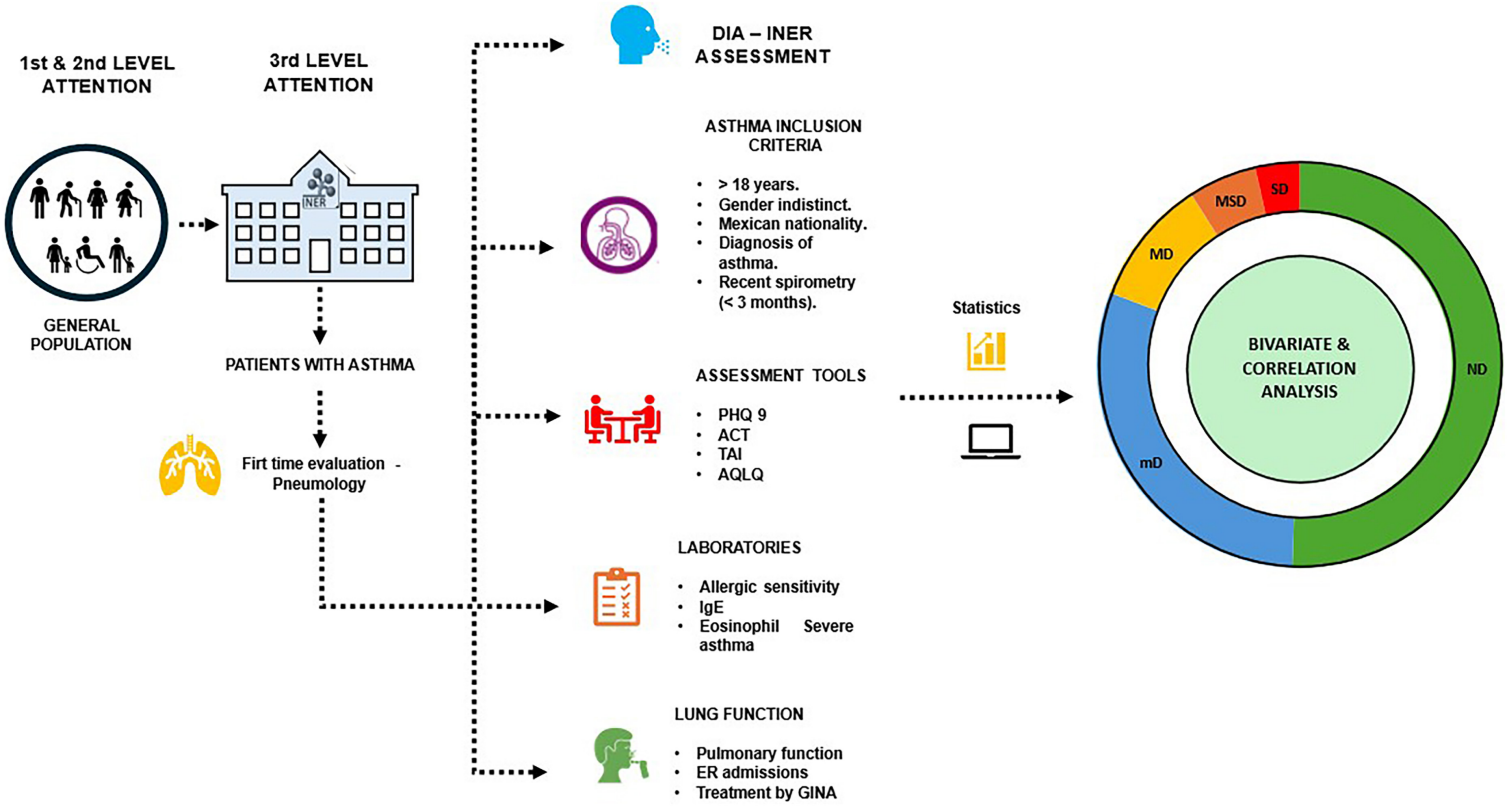
Design of the study. During the first visit to the DIA (Department of Immunogenetic and Allergy), patients with asthma who met the selection criteria underwent assessment using the PHQ-9 questionnaire (Patient Health questionnaire-9/depression), ACT (Asthma Control Test), TAI (Test of Adherence to Inhalers), AQLQ (Asthma Quality of Life Questionnaire), as well as biomarker analysis and lung function test. These items were compared between patients with and without depression using bivariate and correlation analyses.

All patients had been evaluated previously at the pneumology department. At their first allergy consultation, we assessed asthma symptoms using the Asthma Control Test (ACT), treatment adherence using the Test of Adherence to Inhalers (TAI), and lung function using spirometry. Additionally, we analyzed the use of inhaled corticosteroids (ICSs), the use of β-2 agonists, allergy sensitivity, eosinophil cell count, and IgE levels. During the allergy consultation, we administered the Patient Health Questionnaire-9 (PHQ-9) to evaluate depressive symptoms as well as the Asthma Quality of Life Questionnaire (AQLQ) to examine quality of life. In the present study, pregnant patients and patients who did not meet the aforementioned criteria and those who did not complete the questionnaires, clinical biomarker assessments, or pulmonary function tests were excluded from the study (Figure 1).

### 2.2 Lung function

Lung function was assessed using a Jaeger-Care fusion spirometer (Leibnizstrasse, Germany), and prebronchodilator lung function was assessed following the American Thoracic Society guidelines (Pérez-Padilla et al., 2006). A minimum of three maneuvers were performed until quality grade A was achieved, and the best result in terms of the forced expiratory volume in 1 s (FEV1) and forced vital capacity (FVC) parameters was chosen and compared with the predicted values in the Mexican population according to Pérez-Padilla (Pérez-Padilla et al., 2006). Moreover, 15 min after the administration of 400 μg of salbutamol, we evaluated postbronchodilator lung function.

### 2.3 Allergy biomarkers

Allergy sensitization, eosinophil counts, and IgE levels: Allergy sensitization was evaluated through a skin prick test. The test included 40 allergens (Alk-abello; Massachusetts, United States). We expressed the test result as a categorical variable according to whether the result was monosensitivity (1 allergen-positive) or polysensitivity (>1 allergen). The total IgE levels (Architect i2000, Roche, Germany) and eosinophil counts in the blood were measured through hematic cytometry (Beckman Coulter LH750, United States), and the results are expressed as continuous variables.

### 2.4 Asthma control

Asthma control was assessed using the Asthma Control Test (ACT). This test consists of five questions assessing activity limitation, shortness of breath, the presence of nocturnal symptoms, the use of rescue medications, and the patient’s self-report of symptom control in the past 4 weeks. The total score was 25 (range, 5–25), with scores of 20–25, 16–19, and 5–15 indicating well-control, partial control, and poor asthma control, respectively. We used the Spanish version of ACT that was validated in the Mexican population (Schatz et al., 2006; Supplementary Table 1).

### 2.5 Asthma treatment

Asthma treatment was prescribed according to the GINA 2022 guidelines, which aim at the appropriate management and control of asthma. We divided the treatments according to the dose of inhaled corticosteroids (ICSs): low, medium, and high (asthma severity). Additionally, we evaluated the use of β2-agonists (Reddel et al., 2022; Supplementary Table 2).

### 2.6 Therapeutic adherence

Adherence to asthma treatment: Adherence to asthma treatment was examined using the test of adherence to inhalers (TAI). TAI is designed for patients with asthma and/or chronic obstructive pulmonary disease, and it assesses the intensity of adherence, whether good, fair, or poor. TAI consists of 10 questions with scores ranging from 1 to 5 (worst to best adherence), respectively. We classified the results according to the final score: scores of 50, 46–49, and <45 represented good adherence, fair adherence, and poor adherence, respectively (Plaza et al., 2016). We used the Spanish version validated in the Mexican population (see Supplementary Table 3).

### 2.7 Quality of life

Asthma Quality of Life Questionnaire: Quality of life was evaluated using the AQLQ, which was translated and validated in Spanish. It is a 32-item questionnaire. Items are scored from 1 (completely limited) to 7 (not limited). The scale has four dimensions: activities (Items 1, 2, 3, 4, 5, 11, 19, 25, 28, 31, 32), symptoms (Items 6, 8, 10, 12, 16, 18, 20, 22, 24, 29, 30), emotions (Items 7, 13, 15, 21, 27), and environment (Items 9, 17, 23, 25). A higher score on the test was indicative of a better quality of life (Reddel et al., 2022). We used the Spanish version validated in the Mexican population (Supplementary Table 4).

### 2.8 Depression evaluation

Depressive symptom assessment: The PHQ-9 was used to assess the presence of nine diagnostic criteria for depression according to the Diagnostic and Statistical Manual of Mental Disorders-IV (DSM-IV) (American Psychiatric Association (APA), 1994), considering the past 2 weeks. For each criterion, a 4-point scale is used to rate the presence of symptoms, ranging from 0 for “any day” to 3 for “almost every day.” The scores of >5 points were considered suggestive of depression condition (mild depression), 10, 15, and 20 points indicate moderate, moderately severe, and severe depression, respectively. We used the Spanish version validated in the Mexican population (Supplementary Table 5). We used the two-dimensional structure of the PHQ-9: For somatic depression, the items sleep disturbance, fatigue, appetite changes, and psychomotor disturbances were combined, and the total summed score ranged from 0 to 12. For cognitive-affective depression, the items anhedonia, depressed mood, low self-esteem, concentration problems, and suicidal ideation were combined, and the total summed score ranged from 0 to 15 (Supplementary Table 5). All evaluations were performed during the first allergology consultation, which was performed from 2023 to 2024.

### 2.9 Statistics

The required sample size was calculated to detect correlations with *r* = 0.03 between the PHQ-9 and AQLQ, using a two-tailed test, α = 0.05, and 80% power, resulting in a sample size of 82, as determined using the GPower program. A second analysis was conducted using Select Statistical Services. The PHQ-9 scores obtained from a similar sample of patients with asthma (Robinson et al., 2021) showed a mean score of 10.79; considering a 95% confidence level, 80% power, a mean difference of 7.15, and a population variance of 300, the sample size is 92 patients. This implies that our sample (*n* = 102) is adequate for the study. Descriptive statistics were used for demographic variables, frequencies and percentages for categorical variables, and medians and interquartile ranges for continuous variables. Comparative analysis was performed between participants with and without depression. Bivariate associations were determined through Spearman correlation analysis of depressive symptoms, quality of life, lung function, allergy sensitization, asthma control, treatment, and adherence. Analyses were performed using the Statistical Package for the Social Sciences v.21 (SPSS software, IBM, New York, United States). For all comparisons, we considered a *p*-value of <0.05 to indicate statistical significance. Multivariate linear regression models were performed.

### 2.10 Ethics

The present study was reviewed and approved by the Bioethics and Science Committee in Research (protocol B12-21) of the National Institute of Respiratory Diseases, Ismael Cosío Villegas, which complies with the Declaration of Helsinki. Additionally, all patients signed an informed consent form for medical care at the institution (INER-CEECL-01-07-2012) and the institutional form for the protection of personal data (INER-TS-14-01-2013).

## 3 Results

### 3.1 Demographic and clinical data

A total of 102 patients participated in this study. 67 were female and 35 males. The median of age in our sample was 44 years-old. Patients had a mean body mass index of 27.2. In addition, 72.5% (74/102) of patients were classified as being overweight, and 71.5% (73/102) had at least one comorbidity. Specifically, 84.3% (86/102) of patients had allergic rhinitis with a polysensitized pattern. Asthma patients had high eosinophil counts of 250 cells/mm^3^ and IgE levels of 130 IU/L. Additionally, a large proportion of patients had a low socioeconomic status [81/102 (79.4%)].

All asthma patients enrolled had been experiencing asthma symptoms for nearly 2 years. At the first evaluation, they had a score of 21.5 on the ACT, and 82.3% (84/102) required the use of high doses of ICS. Patients exhibited a TAI score of 48. Asthma patients reported having visited the emergency room at least twice during the previous 6 months. Despite these characteristics, they maintained a high score for quality of life according to the AQLQ (with a score of 186) and demonstrated adequate lung function at baseline and postbronchodilator maneuvers (Table 1).

**TABLE 1:** Clinical and demographic data.

Variable	Total	Depression	Non-depression	*p*
N	102	50 (49)	52 (50.9)	
Age	44 (27–56)	44 (25–52.5)	48 (28–57.7)	0.45
Female n (%)	67 (65.6)	33 (66)	34 (65.3)	0.94
SE-low grade n (%)	81 (79.4)	42 (84)	49 (71.1)	0.11
Weight	69.2 (60.3–81.4)	71.2 (61–81.4)	68.1 (60–81.7)	0.6
Height	1.61 (1.55–1.65)	1.61 (1.54–1.68)	1.61 (1.55–1.65)	0.66
BMI	27.2 (24–30.4)	27.7 (24.6–27.7)	26.6 (23.5–30.3)	0.34
Allergic rhinitis n (%)	86 (84.3)	42 (84)	44 (84.6)	0.93
Comorbidities n(%)	73 (71.5)	37 (74)	36 (69.2)	0.59
Eosinophils cells/mm^3^ m(IQR)	250 (92.5–485)	320 (105–655)	215 (52.5–350)	0.1
IgE IU/L levels	130 (28.2–331)	182 (44–331)	96 (12.5–332.7)	0.59
Allergens polysensitivity n (%)	94 (92.1)	46 (91.2	47 (90.3)	1
Years of asthma evolution	2.1 (1.4–8.1)	2.1 (1.4–7.1)	2 (1.2–8.5)	0.95
ACT	21.5 (19–24)	21 (17–23.5)	23 (20–24)	0.06
GINA medium-high doses n(%)	84 (82.3)	46 (92)	44 (84.6)	0.24
TAI	48 (44–50)	46 (43–50)	49 (47–50)	0.01
FVC pre-BD	3.4 (2.7–4.5)	3.3 (2.7–4.5)	3.46 (2.76–4.52)	0.89
FEV1 pre -BD	2.6 (1.9–3.4)	2.6 (2.05–3.52)	2.66 (1.91–3.33)	0.54
FEV1/FVC-BD	77.1 (68.6–83.4)	78 (69.4–83.6)	76.2 (67.1–83.4)	0.79
FVC post -BD	3.25 (2.7–4.3)	3.19 (2.74–4.35)	3.43 (2.71–4.33)	0.87
FEV1 post-BD	2.82 (2.0–3.6)	2.82 (2.11–3.79)	2.86 (2.04–3.47)	0.63
FEV1/FVC post-BD	78.6 (72.4–84.7)	78.7 (73.2–85.1)	77.4 (71–84.2)	0.52
AQLQ score	186 (152.2–209)	174 (144–198)	198 (170–214)	0.01
AQLQ -Symptoms	69.5 (31.7–81)	60 (13–80)	77 (60.5–82)	0.012
AQLQ- Activity	65 (34.5–73)	57 (13.7–69.5)	68 (47.5–75)	0. 012
AQLQ- Emotion	30 (12.7–35)	24 (0–34)	33.5 (27–35)	0.002
AQLQ- Environmental	22 (8.5–26)	18.5 (0–26)	23.5 (13.5–27)	0.03
PHQ-9 score	4.5 (2–8)	8 (5.7–12.5)	2 (1.25–4)	0.001
Cognitive symptoms	2 (1–3)	3 (2–5.2)	1 (0–2)	0.001
Somatic symptoms	1.5 (0–3)	3 (2–5)	1 (0–1)	0.001

BMI, Body mass index; IgE, Immunoglobnulin E; ACT, Asthma Control Test. The asthma treatment was prescribed according to the Global Initiative for Asthma-GINA- 2022 guidelines; FVC, Forced Vital Capacity; FEV_1,_ Forced Expiratory Volume in the first second; pre-BD, pre-bronchodilatador; postBD, post-bronchodilatador; TAI, Test of Adhesion to Inhalers; AQLQ, Asthma Quality of Life Questionnaire; PHQ-9, Patient Health Questionnaire-9.

**FIGURE 2:**
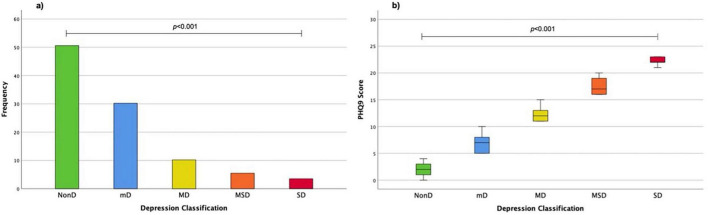
**(a)** Distribution of depression condition among patients with asthma. **(b)** PHQ-9 score among patients with asthma. NonD, non depression; mD, mild depression; MD, moderate depression; MSD, moderately severe depression; SD, severe depression.

According to the PHQ-9 score, nearly half of the asthma patients experienced some degree of depression [50/102 (49%)]. Most patients had mild depression [33/102 (32.3%)], whereas only a small percentage [6/102 (5.8%)] of patients were classified as having severe depression (*p* < 0.001) (Figure 2a). Fifty percent of asthma patients had scores >5 on the PHQ-9, indicating the presence of depression. Asthma patients with depression had higher scores on both the cognitive and the somatic components of the PHQ-9 (*p* = 0.01) (Figure 2b).

A bivariate analysis showed that asthma patients without depression, exhibited higher score [adherence (*p* = 0.01), quality of life in all its domains] compared with those with depression (*p* < 0.03), as well a greater clinical control of asthma (*p* = 0.06); no other difference was found (Table 1).

### 3.2 Analysis of correlation

A moderate negative correlation of the PHQ-9 score was noted with asthma control, as assessed using the ACT (*r*s = −0.36, *p* < 0.001), and a moderate positive correlation with GINA treatment steep (*r*s = 0.41, *p* < 0.001). These findings suggest that higher depressive symptoms are associated with poorer asthma control as measured by ACT as well as higher treatment intensity. The ACT score was moderately and consistently related to better quality of life (total AQLQ: *r* = 0.50, *p* <0.001; domains: *r* = 0.39–0.52), demonstrating that better asthma control is related to better quality of life. Similarly, the increased TAI score was negatively correlated with the PHQ score (*r* = −0.36; *p* < 0.001) and positively correlated with the AQLQ score (*r* = 0.26; *p*<0.001). This finding suggests that higher PHQ scores are associated with lower treatment adherence and, in turn, with worse quality of life. In most comparisons, no relevant correlations were observed between depression or quality of life scores and biomarkers (eosinophils, IgE) or lung function parameters, except for a correlation trend with baseline FVC (Table 2).

**TABLE 2:** Correlation analysis.

Variable	Somatic symptoms	Cognitive/affective symptoms	PHQ 9 total	AQLQ symptoms	AQLQ activity limitation	AQLQ emotion	AQLQ environment	AQLQ total
Lung function								
FVC pre	−0.10	−0-06	**0.27**	−0.01	−0.07	−0.06	−0.12	−0.06
FEV pre	0.08	0.15	−0.002	−0.001	0.034	0.02	0.02	0.019
FEV1/FVC pre	0.18	0.15	−0.11	0.08	0.12	0.10	0.15	0.11
FVC post	0.03	0.04	−0.11	−0.01	0.025	0.008	0.028	−0.018
FEV1 post	0.041	0.14	0.004	−0.02	0.001	0.001	0.018	−0.005
Biomarkers								
Eosinophils count	0.10	−0.05	0.02	0.06	0.01	0.05	−0.01	0.03
IgE	0.05	0.20	0.06	0.09	0.05	0.09	0.11	0.08
Allergy sensitization	−0.003	0.11	0.08	0.18	0.21	0.18	0.16	0.19
Asthma control, treatment, and adherence								
ACT total	0.045	0.05	−0.36	0.52	0.46	0.39	0.45	0.50
GINA control	0.23	0.15	0.41	−0.22	−0.12	−0.23	−0.16	−0.19
TAI Total	0.038	−0.17	−0.36	0.27	0.22	0.23	0.25	0.26

FVC, Forced Vital Capacity; FEV_1_, Forced Expiratory Volume in 1 Second; pre-BD, pre-bronchodilatador, post-BD, post bronchodilatador: 15 min after the administration of 400 μg of salbutamol. ACT, Asthma Control Test; The asthma treatment was prescribed according to the GINA-Global Initiative for Asthma-2022 guidelines, TAI, Test of Adhesion to Inhalers; AQLQ, Asthma Quality of Life Questionnaire. *p* ≤ 0.05, *p* ≤ 0.01, *p* ≤ 0.001.

### 3.3 Multivariable linear regression models

Two multivariable linear regression models were performed. The first model included asthma control (ACT), treatment adherence (TAI), and treatment intensity (GINA). The model was statistically significant [*R*^2^ = 0.257, adjusted *R*^2^ = 0.221, *F*(3, 62) = 7.14, *p*<0.001], explaining 25.7% of the variance in PHQ-9 scores. Among the predictors, only treatment intensity (GINA step) remained independently associated with depressive symptoms (standardized β = 0.308, *p* = 0.021). A second model including physiological parameters (FEV_1_/FVC ratio, eosinophil count, serum IgE levels, and allergic sensitization) was not statistically significant [*R*^2^ = 0.021, adjusted *R*^2^ = −0.055, *F*(4, 51) = 0.277, *p* = 0.891], and none of the physiological variables independently predicted PHQ-9 scores (Table 3).

**TABLE 3:** Summary of the multiple linear regression model for predicting depression scores.

Coefficients	Unstandarized	Standarized	*t*	*p*
Model 1				
TAI	−0.09	−0.13	−1.24	0.21
GINA	2.01	0.30	2.37	0.02
ACT	−0.28	−0.22	−1.71	0.09
Model 2				
Eosinophils count	1.45 × 10^–5^	0.015	0.108	0.91
Allergy sensitization	1.12 × 10^–4^	0.118	0.85	0.39

TAI, Test of adhesion to inhalers; GINA, Global initiative on asthma outcomes (treatment); ACT, asthma control test, IgE, Immunoglobulin E.

## 4 Discussion

In this study our findings support the growing evidence that psychological factors are closely intertwined with asthma management in routine clinical practice. In this real-world sample depression were associated with poorer asthma-related outcomes, reinforcing the view that asthma should be understood not only as a chronic inflammatory disease but also as a condition substantially influenced by psychological wellbeing and quality of life.

A significant finding is that a large proportion of patients evaluated in our study had depression according to the PHQ-9 instrument. This finding is consistent with the reports by Opolski and Wilson (2005) and Bedolla-Barajas et al. (2021), who reported that 45–50% of patients with asthma have depressive symptoms or major depressive episodes associated to asthma symptoms.

In relation to depressive symptoms, the negative relationships with asthma control and adherence to ICS indicate that greater adherence to these drugs and better subjective control of symptoms of asthma are related to lower depressive symptoms. Similarly, a tendential correlation was also observed between lung function and depression, such as between FVC and the total depression score. Findings on the relationship between asthma control and depression have been reported in the literature. Ciprandi et al. (2015) reported a correlation between patient-reported asthma control and depressive symptoms, but no relationship of objective adherence with treatment was observed. These data led the authors to propose a possible explanation for this phenomenon; depression intensifies the perception of asthma symptoms. Controlled clinical trials have reported that the use of inhaled corticosteroid therapy and beta agonists improves lung function and asthma control after 3 months. However, in the real-life setting, although therapeutic adherence was not optimal in the population, the use of ICS demonstrated therapeutic efficacy. This has been demonstrated in patients receiving treatment for chronic diseases such as asthma, whose therapeutic adherence is around 50% (Pan American Health Organization, 2004; Santibáñez et al., 2023).

The proposal is interesting considering that in this study, no significant correlations were identified between depression and lung function. The results of multivariable linear regression suggest that depressive symptoms are more closely associated with treatment intensity than with objective physiological markers of asthma severity or allergic inflammation. The absence of independent associations with eosinophil count, serum IgE, allergic sensitization, or lung function supports the hypothesis that psychological burden in asthma may be influenced more by the clinical experience of living with the disease than by biological indicators alone. In a study of patients with poor asthma control, Mangold et al. (2018) reported the same finding; specifically, lung function was not associated with depressive symptoms. In principle, these findings could indicate that, at least, a part of the variance in the depression phenomenon in patients with asthma is related to the subjects’ perceived ability to control their disease. In the same study by Mangold et al. (2018), patients with depression and poor asthma control had less knowledge about the disease and self-efficacy in its treatment. The discrepancy between the perceived burden of disease, especially in the presence of depressive symptoms, and the objective expression of asthma suggests that many patients report worse control, greater dyspnea and poorer quality of life even when physiological parameters remain stable; findings observed in our research regarding lung function and/or eosinophil levels in both comparison groups. This evidence shows that depression could amplify symptom perception through mechanisms such as somatic hypervigilance and negative interpretation of bodily sensations, meaning that subjective questionnaires may reflect emotional distress rather than inflammatory activity (Steele et al., 2012). This has clinical implications for the need to interpret patient reported outcomes carefully, to systematically screen for depression, and to integrate multimodal interventions that address the biological and emotional dimensions of asthma (Benfante et al., 2023).

However, the correlation between FVC and high GINA treatment steps and depression suggest that asthma severity may influence patients’ mood. Importantly, reduced lung function can significantly impair physical health and overall health, although the relationship we found with this measure was weak. Choi et al. (2017) reported lower scores for forced expiratory volume, forced vital capacity, and their ratio in patients with asthma and depression. The authors propose that for these conditions, a common pathway is alterations in the autonomic nervous system.

It has been widely documented that asthma patients report alterations in their quality of life (Juniper et al., 1999; Rönnebjerg et al., 2024; Sastre et al., 2018; Sundbom et al., 2016). The data from our study indicate a specific relationship between QOL and subjective asthma control, which has been observed previously (Juniper et al., 1999; Rönnebjerg et al., 2024). It was also possible to identify a relationship between quality of life according to AQLQ and adherence to inhalers by TAI. These data contrast with the previous findings by Ciprandi et al. (2015). This discrepancy may be due to differences in the methodology used to measure adherence. Renzi used the medication possession ratio (MPR), whereas in our study, we used self-reports with the Test of Adherence to Inhalers. Probably, each patient’s personal experience of control and adherence to treatment plays fundamental roles in the subjective experience of quality of life and emotional distress.

This point can also be supported by the absence of a relationship between lung capacity measurements and asthma control based on the GINA score with quality of life in our study, which has been consistently described previously (Colombo et al., 2019; Juniper et al., 1999). Studies have suggested that rather than objective lung function, other factors comorbid with asthma, such as obesity, waist circumference (Colombo et al., 2019), or sleep quality (Rönnebjerg et al., 2024), influence specific domains of quality of life.

Taken together, our findings suggest that depressive symptoms are associated with symptom severity on the one hand and with the subject’s perception of control over their medical condition and adherence to treatment on the other hand. The development of depressive symptoms can be attributed to two causes: the first being the inflammatory involvement of both asthma and endogenous depression, and the second to factors such as coping skills, agency, and self-control, which are related to emotional distress. Quality of life may be more specifically related to the experience of disease control and adherence to treatment, but not necessarily to lung function or depression as a comorbidity accompanying asthma (Supplementary Material).

The findings of our research support the use of a psychoeducational or cognitive-behavioral intervention that focuses on teaching patients about their condition. These interventions help patients understand their condition better, learn how to cope with it, and adhere to treatment recommendations better. Furthermore, such interventions lead to improvements in the patient’s financial situation and positive effects on the overall wellbeing and quality of life of their immediate family by reducing complications, treatment-related costs, and the burden of care. In this context, it becomes essential to position the patient as an active participant in managing their condition and promoting their informed and responsible involvement in the health–illness process, which directly contributes to strengthening their autonomy and optimizing their quality of life.

This study has several limitations. First, it was conducted at a single center, which may limit the generalizability of the findings. Although the study was performed at the National Institute of Respiratory Diseases (INER), the national referral center for respiratory diseases in Mexico, multicenter recruitment was not feasible because the study was carried out during the period following the COVID-19 pandemic. Replication in other clinical settings and healthcare systems is therefore warranted.

Second, depression and several asthma-related outcomes were assessed using self-report instruments. Although the PHQ-9 does not replace a structured clinical interview for diagnosing depression, it has demonstrated excellent psychometric properties, including high internal consistency and good agreement with established measures such as the Beck Depression Inventory-II in pretreatment populations (α = 0.86) (Titov et al., 2011). Moreover, its brevity, ease of administration, free availability, and suitability for routine clinical screening make it particularly useful in real-world healthcare settings (Kroenke et al., 2001; Spitzer et al., 1999). Likewise, measures such as the ACT, TAI, and AQLQ capture the patient’s subjective experience and may therefore be influenced by individual perceptions.

However, this subjectivity should not be regarded solely as a source of measurement bias. Our findings suggest that patients’ perceptions of their disease, treatment adherence, and quality of life may themselves be influenced by depressive symptoms, highlighting an important psychological dimension of asthma management. Future studies combining patient-reported outcomes with objective measures of asthma control, treatment adherence, and clinical status could help disentangle the relative contribution of subjective perceptions and objective disease characteristics.

Finally, the cross-sectional design precludes conclusions regarding the temporal or causal relationships among depression, asthma control, treatment adherence, and quality of life. Longitudinal studies incorporating both objective clinical assessments and repeated psychological evaluations will be necessary to clarify these relationships and to determine whether interventions targeting depression improve asthma-related outcomes over time.

## 5 Conclusion

This study analyzed the association between asthma and mood disorders. More than half of the participants experienced depressive symptoms that adversely affected their wellbeing. Subjective asthma control and self-reported adherence are the strongest predictors of quality of life and lower emotional distress. Objective measures such as lung function and allergy sensitization are minimally associated with mood. This result indicates the importance of patient-perceived agency in shaping mental health outcomes. Furthermore, both the somatic and the cognitive-affective aspects of depression substantially hinder specific areas of quality of life, especially symptoms and emotional functioning. Consequently, integrating respiratory and psychiatric screening through a multidisciplinary approach is vital to address the combined challenges of chronic inflammation and psychological comorbidities. Further studies are necessary to explore these findings and their implications for future therapeutic approaches for patients with asthma.

## Data Availability

The original contributions presented in this study are included in the article/Supplementary material, further inquiries can be directed to the corresponding authors.
