# Supplementary material for: Association of asthma control, treatment, adherence, lung function, and allergy sensitization with depressive symptoms and quality of life in a real-life setting
Source: Front Psychol. 2026 Jul 31;17:1882738. doi: 10.3389/fpsyg.2026.1882738 (PMC13473153; doi:10.3389/fpsyg.2026.1882738)
Supplement: Supplementary file 1 [file Supplementary_file_1.DOCX]

**Supplementary Material**

**Supplementary Table 1. Asthma Control**

| Asthma control test | | | | | Score |
| --- | --- | --- | --- | --- | --- |
| 1. In the past 4 weeks, how much of the time did you asthma keep you from getting as much done at work, school or at home? | | | | | 1-5 |
| All of the time (1) | Most of the time (2) | Some of the time (3) | A little of the time (4) | None of the time (5) |  |
| 2. During the past 4 weeks, how often have you had shortness of breath? | | | | | 1-5 |
| More than once a day (1) | Once a day (2) | 3 to 6 times a week (3) | Once or twice a week (4) | Not at all (5) |  |
| 3. During the past 4 weeks, how often did your asthma symptoms (wheezing, coughing, shortness of breath, chest tightness or pain) wake you up at night or earlier than usual in the morning? | | | | | 1-5 |
| 4 or more nights a week (1) | 2 to 3 nights a week (2) | Once a week (3) | Once or twice (4) | Not at all (5) |  |
| 4. During the past 4 weeks, how often have you used your rescue inhaler or nebulizer medication (such as albuterol)? | | | | | 1-5 |
| 3 or more times per day (1) | 1 or 2 times per day (2) | 2 or 3 times per week (3) | Once a week or less (4) | Not at all (5) |  |
| 5. How would you rate your asthma control during the past 4 weeks? | | | | | 1-5 |
| Not controlled at all (1) | Poorly controlled (2) | Somewhat controlled (3) | Well controlled (4) | Completely controlled (5) |  |

**Supplementary Table 2. Asthma treatment according to GINA guidelines 2023**.

| **Drug** | **Daily dose (mcg)** | | |
| --- | --- | --- | --- |
|  | **Low** | **Medium** | **High** |
| Beclometasone dipropionate (pMDI, standard particle, HFA) | 200-500 | >500-1000 | >1000 |
| Beclometasone dipropionate (HFA, DPI or pMDI, extrafine) | 100-200 | >200-400 | >400 |
| Budesonide (DPI, pMDI, standard particle, HFA) | 200-400 | >400-800 | >800 |
| Ciclesonide (HFA, pMDI, extrafine particle) | 80-160 | >160-320 | >320 |
| Fluticasone furoate (DPI) | 100 | | 200 |
| Fluticasone propionate (DPI) | 100-250 | >250-500 | >500 |
| Fluticasone propionate (pMDI, standard particle, HFA) | 100-250 | >250-500 | >500 |
| Mometasone furoate (DPI) | Depends on DPI device – see product information | | |
| Mometasone furoate (pMDI, standard particle, HFA) | 200-400 | | >400 |

HFA – hydrofluoroalkane propellant; pMDI – pressurized metered-dose inhaler.

**Supplementary Table 3. Test of Adherence to Inhalers (TAI) Questionnaire**

| Test of Adherence to Inhalers (TAI) Questionnaire | | | | | Score |
| --- | --- | --- | --- | --- | --- |
| 1. During the last 7 days, how many times did you forge to take your usual inhalers? | | | | | 1 to 5 |
| All (1) | More than half (2) | Approximately a half (3) | Less than half (4) | None (5) |  |
| 2. Do you forge to take inhalers? | | | | | 1 to 5 |
| Always (1) | Mostly (2) | Sometimes (3) | Rarely (4) | Never (5) |  |
| 3. When you feel good about you illness, do you stop taking your inhalers? | | | | | 1 to 5 |
| Always (1) | Mostly (2) | Sometimes (3) | Rarely (4) | Never (5) |  |
| 4. When you are on vacation or weekend, do you stop taking your inhalers? | | | | | 1 to 5 |
| Always (1) | Mostly (2) | Sometimes (3) | Rarely (4) | Never (5) |  |
| 5. When you are nervous or sad, do you stop taking your inhalers? | | | | | 1 to 5 |
| Always (1) | Mostly (2) | Sometimes (3) | Rarely (4) | Never (5) |  |
| 6. Do you stop taking your inhalers because of fear of side effects? | | | | | 1 to 5 |
| Always (1) | Mostly (2) | Sometimes (3) | Rarely (4) | Never (5) |  |
| 7. Do you stop taking your inhalers because of considering they are useless to treat your condition? | | | | | 1 to 5 |
| Always (1) | Mostly (2) | Sometimes (3) | Rarely (4) | Never (5) |  |

**Supplementary Table 4. Asthma Quality of Life Questionnaire (AQLQ).**

| **Supplementary Table 4. Asthma Quality of Life Questionnaire (AQLQ)** | | | | | | | |
| --- | --- | --- | --- | --- | --- | --- | --- |
| **QUALITY OF LIFE QUESTIONNAIRE FOR ASTHMATIC PATIENTS WITH STANDARDIZED ACTIVITIES (AQLQ(S))** | | | | | | | |
|  | | | | | | | |
| **Self administered. Spanish version for Mexico (≥12 years)** | | | | | | | |
| Please answer all questions by circling the number that best describes how you have been during the last 2 weeks because of your asthma | | | | | | | |
| How limited have you been during the past 2 weeks from doing the following activities because of your asthma? | Totally limited | Extremely limited | Very limited | With moderate limitation | With some limitation | With little limitation | Nothing limited |
| 1. Strenuous activities (such as rushing, exercising, running up stairs, playing sports) | 1 | 2 | 3 | 4 | 5 | 6 | 7 |
| 2. Moderate activities (such as walking, doing chores, gardening, shopping, climbing stairs) | 1 | 2 | 3 | 4 | 5 | 6 | 7 |
| 3. Social activities (such as talking, playing with pets/children, visiting friends/relatives) | 1 | 2 | 3 | 4 | 5 | 6 | 7 |
| 4. Activities related to your work or school (work you have to do at work or school)  If you are not employed or self-employed, refer to the tasks you must perform almost every day | 1 | 2 | 3 | 4 | 5 | 6 | 7 |
| 5. Sleep | 1 | 2 | 3 | 4 | 5 | 6 | 7 |
| How much physical or emotional discomfort have you felt during the past 2 weeks? | Enormously | Much | Pretty much | Regular | A little bit | Next to nothing | Nothing |
| 6. How much physical or emotional discomfort have you felt during the past 2 weeks because of the tightness in your chest? | 1 | 2 | 3 | 4 | 5 | 6 | 7 |
| In general, how long during the last 2 weeks? | Always | Almost always | Much of the time | Part of the time | Short time | Almost never | Never |
| 7. Have you felt worried because you have asthma? | 1 | 2 | 3 | 4 | 5 | 6 | 7 |
| 8. Have you felt short of breath due to asthma? | 1 | 2 | 3 | 4 | 5 | 6 | 7 |
| 9. Have you had asthma symptoms from exposure to cigarette smoke? | 1 | 2 | 3 | 4 | 5 | 6 | 7 |
| 10. Has there been a hissing or whistling sound in your chest? | 1 | 2 | 3 | 4 | 5 | 6 | 7 |
| 11. Did you feel like you had to avoid a situation or place because of cigarette smoke? | 1 | 2 | 3 | 4 | 5 | 6 | 7 |
| How much physical or emotional discomfort have you felt during the past 2 weeks? | Totally limited | Extremely limited | Very limited | With moderate limitation | With some limitation | With little limitation | Nothing limited |
| 12. How much physical or emotional discomfort have you felt during the last 2 weeks from the cough? | 1 | 2 | 3 | 4 | 5 | 6 | 7 |
| In general, how long during the last 2 weeks...? | Always | Almost always | Much of the time | Part of the time | Short time | Almost never | Never |
| 13. Have you been frustrated by asthma? | 1 | 2 | 3 | 4 | 5 | 6 | 7 |
| 14. Have you felt heaviness in your chest due to asthma? | 1 | 2 | 3 | 4 | 5 | 6 | 7 |
| 15. Have you been concerned about the need to use asthma medication? | 1 | 2 | 3 | 4 | 5 | 6 | 7 |
| 16. Have you felt the need to clear your throat and remove phlegm? | 1 | 2 | 3 | 4 | 5 | 6 | 7 |
| 17. Have you had asthma symptoms from exposure to dust? | 1 | 2 | 3 | 4 | 5 | 6 | 7 |
| 18. Have you had difficulty breathing in because of asthma? | 1 | 2 | 3 | 4 | 5 | 6 | 7 |
| 19. Did you feel that you had to avoid a situation or place because of the dust? | 1 | 2 | 3 | 4 | 5 | 6 | 7 |
| 20. Have you woken up in the morning with asthma symptoms? | 1 | 2 | 3 | 4 | 5 | 6 | 7 |
| 21. Have you been afraid you won't have your asthma medication on hand? | 1 | 2 | 3 | 4 | 5 | 6 | 7 |
| 22. Has shortness of breath bothered you? | 1 | 2 | 3 | 4 | 5 | 6 | 7 |
| In general, how long during the last 2 weeks...? | Always | Almost always | Much of the time | Part of the time | Short time | Almost never | Never |
| 23. Have you had asthma symptoms from weather or pollution? | 1 | 2 | 3 | 4 | 5 | 6 | 7 |
| 24. Have you been woken up by asthma at night? | 1 | 2 | 3 | 4 | 5 | 6 | 7 |
| 25. Have you avoided going out or limited your outings due to weather or pollution? | 1 | 2 | 3 | 4 | 5 | 6 | 7 |
| 26. Have you had asthma symptoms from exposure to strong odors or perfumes? | 1 | 2 | 3 | 4 | 5 | 6 | 7 |
| 27. Have you been afraid of running out of air or breathing? | 1 | 2 | 3 | 4 | 5 | 6 | 7 |
| 28. Did you feel that you had to avoid a situation or place because of strong smells or perfumes? | 1 | 2 | 3 | 4 | 5 | 6 | 7 |
| 29. Has asthma prevented you from sleeping well? | 1 | 2 | 3 | 4 | 5 | 6 | 7 |
| 30. Have you ever felt like you're struggling to breathe? | 1 | 2 | 3 | 4 | 5 | 6 | 7 |
| How much has asthma limited you during the past 2 weeks? | Most | Many | Several | Some | Very few | Almost none | None (i.e. I've done all the activities I wanted) |
| 31. Think about all the activities you would have liked to do during the last 2 weeks.  How many of these activities have you stopped doing because of asthma? | 1 | 2 | 3 | 4 | 5 | 6 | 7 |
| 32. Overall, of all the activities you have done during the last 2 weeks,  How much has asthma limited you? | 1 | 2 | 3 | 4 | 5 | 6 | 7 |

**Supplementary Table 5. Patient Health Questionnaire-9 (PHQ-9)**

| Over the last 2 weeks, how often have you been bothered by any of the following problems? (Use “✔” to indicate your answer) | Not at all | Several days | More than half the days | Nearly every day |
| --- | --- | --- | --- | --- |
| 1. Little interest or pleasure in doing things | 0 | 1 | 2 | 3 |
| 2. Feeling down, depressed, or hopeless | 0 | 1 | 2 | 3 |
| 3. Trouble falling or staying asleep, or sleeping too much | 0 | 1 | 2 | 3 |
| 4. Feeling tired or having little energy | 0 | 1 | 2 | 3 |
| 5. Poor appetite or overeating | 0 | 1 | 2 | 3 |
| 6. Feeling bad about yourself — or that you are a failure or have let yourself or your family down | 0 | 1 | 2 | 3 |
| 7. Trouble concentrating on things, such as reading the newspaper or watching television | 0 | 1 | 2 | 3 |
| 8. Moving or speaking so slowly that other people could have noticed? Or the opposite — being so fidgety or restless that you have been moving around a lot more than usual | 0 | 1 | 2 | 3 |
| 9. Thoughts that you would be better off dead or of hurting yourself in some way | 0 | 1 | 2 | 3 |
